# Deterioration in saliva quality in patients with Sjögren’s syndrome: impact of decrease in salivary epidermal growth factor on the severity of intraoral manifestations

**DOI:** 10.1186/s41232-018-0062-0

**Published:** 2018-04-09

**Authors:** Naoto Azuma, Yoshinori Katada, Hajime Sano

**Affiliations:** 1 Division of Rheumatology, Department of Internal Medicine, Hyogo College of Medicine, 1-1 Mukogawa-cho, Nishinomiya, Hyogo 663-8501 Japan; 2 Division of General Medicine, Department of Internal Medicine, Sakai City Medical Center, 1-1-1 Ebaraji-cho, Nishi-ku, Sakai, 593-8304 Japan

**Keywords:** Dry mouth, Epidermal growth factor, Intraoral manifestation, Oral mucosal involvement, Quality of life, Saliva, Saliva quality, Sjögren’s syndrome, Xerostomia

## Abstract

**Background:**

Sjögren’s syndrome (SS) is a chronic inflammatory autoimmune disease characterized by lymphocytic infiltration of the exocrine glands, especially the salivary and lacrimal glands. As a result of salivary gland dysfunction, most patients with SS have xerostomia related to a reduced salivary flow rate. In addition to the discomfort due to xerostomia, dry mouth can cause various intraoral manifestations such as refractory stomatitis, ulcer, and atrophic changes in the oral mucosa and tongue, and the patient’s quality of life (QoL) is severely impaired. These manifestations are believed to be caused mainly by a decrease in the clearance in the oral cavity owing to hyposalivation. However, because saliva has several beneficial physiological effects on the intraoral environment, qualitative changes in sialochemistry should also be considered a cause of the refractory intraoral manifestations in SS.

**Main text:**

Salivary epidermal growth factor (EGF) is considered an important cytoprotective factor against injuries. It contributes to wound healing in the oral cavity and to maintenance of mucosal integrity in the oral cavity and gastrointestinal tract. We evaluated changes in salivary EGF levels and assessed the association between salivary EGF levels and the severity of intraoral manifestations in patients with SS. The following novel findings were obtained: (1) salivary EGF levels in SS patients were significantly lower than those in non-SS patients; (2) salivary EGF levels as well as the salivary flow rate decreased with the progression of SS; (3) with prolonged SS disease duration, salivary EGF levels decreased more rapidly than the salivary flow rate; and (4) decreases in salivary EGF levels significantly correlated with exacerbation of the oral health-related QoL in patients with SS.

**Conclusions:**

The deterioration in saliva quality as well as lower intraoral clearance by hyposalivation could play a role in the pathogenesis of refractory intraoral manifestations in patients with SS. Our findings suggest a new target for therapeutic intervention for SS.

## Background

Sjögren’s syndrome (SS) is a chronic inflammatory autoimmune disease characterized by lymphocytic infiltration of the exocrine glands, especially the salivary and lacrimal glands. As a result of salivary gland dysfunction, most patients with SS have xerostomia caused by a reduced salivary flow rate. In addition to the discomfort, xerostomia can cause various intraoral manifestations, for example, dental caries and oral mucosal involvements, such as refractory stomatitis, oral ulcers, and atrophic changes in the oral mucosa and lingual papilla. In the chronic form, these manifestations can severely impair a patient’s quality of life (QoL) [1]. The intraoral manifestations in patients with SS are believed to be caused mainly by decreased clearance in the oral cavity owing to hyposalivation. However, because saliva has several beneficial physiological effects on the intraoral environment, such as lubrication, maintenance of mucosal integrity, and antimicrobial activity [2], qualitative changes in its composition should also be considered as a cause of the refractory intraoral manifestations in SS.

Epidermal growth factor (EGF), which accelerates incisor eruption and eyelid opening in newborn animals, was first isolated from mouse submandibular glands [3]. EGF is a polypeptide comprising 53 amino acids (molecular weight, 6.045 kDa) that promotes the growth of various tissues in several species [4]. In humans, EGF is produced by the salivary glands and duodenal Brunner’s glands [5]. The main source of EGF in the oral cavity is the parotid glands [4, 6]; however, salivary EGF has been found to be secreted not only from the parotid and submandibular glands but also from the sublingual or minor salivary glands [4, 6, 7]. EGF binding to the EGF receptor stimulates the activity of intracellular kinase cascades, producing signals that consequently alter gene regulation, which leads to cell proliferation and anti-apoptogenic survival [8, 9]. Salivary EGF is considered an important cytoprotective factor against injuries, and it contributes to wound healing and maintenance of mucosal integrity in the oral cavity [10, 11] and in the gastrointestinal tract [12]. Although the detailed mechanisms by which EGF secretion into the saliva is controlled are not yet known, several studies have found that salivary EGF levels are significantly decreased in patients with intraoral inflammatory lesions, such as aphthous stomatitis [4, 13] and peritonsillar abscess [4]. In addition, patients with oral mucositis induced by radiation therapy for head and neck carcinoma were also found to have markedly low salivary EGF levels [14, 15]. These findings suggested that low salivary EGF levels reduce the capacity of the oral mucosa to heal after injury and maintain its physiological integrity.

To the best of our knowledge, no study conducted to date has measured salivary EGF levels in patients with SS. Thus, we evaluated changes in salivary EGF levels in patients with SS and assessed the association between salivary EGF levels and the severity of intraoral manifestations in SS [16, 17].

## Assessment of the association between salivary EGF and SS

### Methods

#### Selection of patients

##### To assess changes in the salivary EGF levels in patients with SS

Forty patients with SS (27 primary SS, 13 secondary SS) participated in this study. All patients (SS group) fulfilled the revised Japanese criteria for the diagnosis of SS proposed by the Japanese Ministry of Health and Welfare (1999) [18] and the American-European Consensus Group classification criteria for SS (2002) [19]. Twenty-three individuals without SS, including healthy individuals and those with rheumatoid arthritis, polymyalgia rheumatica, dermatomyositis, bronchial asthma, systemic lupus erythematosus, adult-onset Still’s disease, relapsing polychondritis, synovitis-acne-pustulosis-hyperostosis-osteitis (SAPHO) syndrome, and eosinophilia, were recruited as controls (non-SS group). The non-SS group patients could not be diagnosed with SS and salivary gland dysfunction on the basis of their clinical symptoms, physical findings, and laboratory findings through the clinical course. The exclusion criteria consisted of factors that are known to affect the intraoral environment or saliva secretion and salivary EGF levels and were as follows: current smoking; chronic alcohol use; ongoing dental treatment; recurrent oral mucositis due to conditions other than SS; treatment with antiparkinson drugs or psychiatric drugs such as antidepressants, anti-anxiety agents, and antipsychotic agents; severe diabetes mellitus; severe reflux esophagitis; past history of head and neck carcinoma; previous radiation therapy to the head and neck region; and previous chemotherapy for cancer.

##### To assess changes in the salivary EGF levels at 3-year follow-up in the same patients with SS

Twenty-three patients with SS (14 primary SS, nine secondary SS) and 14 individuals without SS serving as controls (non-SS group) who participated in the above study and were subsequently followed up for 3 years were enrolled in this study.

These studies were approved by the ethics committee of the Hyogo College of Medicine (no. 758). All subjects provided written informed consent to participate in the study.

#### Saliva collection and quantification of salivary EGF

Whole stimulated saliva was collected after the subjects chewed gum (Free Zone Gum Hi-Mint®; Lotte, Tokyo, Japan) for 10 min and expectorated into graduated centrifuge tubes. All samples were similarly collected at approximately the same time before breakfast in the morning, with fasting, because salivary EGF concentrations show apparent changes related to food intake [4]. The final saliva volume was measured, and EGF levels in the supernatants obtained by centrifuging the saliva samples were measured using a commercial enzyme-linked immunosorbent assay kit (Quantikine®; R&D System, Minneapolis, MN, USA). Total salivary EGF output (pg/10 min) was calculated by multiplying salivary EGF concentration (pg/ml) by saliva volume (ml/10 min) [15].

#### Quantitative assessment of intraoral manifestations

At the time of saliva collection, subjective intraoral manifestations were assessed using the short Japanese version of the Oral Health Impact Profile (OHIP) [20, 21], which is a self-administered questionnaire. The OHIP is one of the most widely used instruments to measure oral health-related QoL (OHRQoL). The OHIP-14 consists of 14 questions designed to measure the frequency of problems associated with the teeth, mouth, or dentures. The questions have seven aspects: functional limitation, physical pain, psychological discomfort, physical disability, psychological disability, social disability, and handicap. The answers to the questions were given using a 5-point scale ranging from 0 to 4 (0, never; 1, hardly ever; 2, occasionally; 3, fairly often; and 4, very often). The unweighted scores were subsequently combined to obtain a single summary score with a possible range of 0–56, with a high score indicating more frequent problems, that is, poorer OHRQoL. It has been demonstrated by Stewart et al. that in patients with SS, lower salivary flow rates are significantly associated with poorer oral health, as assessed using the OHIP-14 summary score [1].

#### Statistical analysis

Results are expressed as mean ± standard deviation. The Mann-Whitney *U* test, chi-square test, Fisher’s exact test, or Wilcoxon signed-rank test was used as appropriate to compare differences between groups. The correlations were examined using the Spearman’s rank correlation coefficient. A *p* value < 0.05 was considered statistically significant.

### Results

#### Assessment of changes in the salivary EGF levels in patients with SS

##### Comparison of the clinical data in patients with and without SS

The characteristics of the study groups are presented in Table 1. No significant differences in age and sex were observed between the groups. The mean disease duration of SS was 5.6 years. The salivary flow rate in the SS group (7.8 ± 4.4 mL/10 min) was significantly lower than that in the non-SS group (16.9 ± 5.9 mL/10 min) (*p* < 0.0001). The OHIP-14 score in the SS group (11.3 ± 9.4) was significantly higher than that in the non-SS group (7.1 ± 7.6) (*p* = 0.037). Thus, the OHRQoL of SS patients was poorer than non-SS patients.

**Table 1:** Clinical characteristics of the study groups

	SS (*n* = 40)	Non-SS (*n* = 23)	*p* value
Age (years)	55.4 ± 13.2	56.1 ± 17.4	0.425
Sex (male/female, number)	3:37	5:18	0.129
Disease duration (years)	5.6 ± 3.7 (*n* = 24)	–	–
Salivary flow rate (mL/10 min)	7.8 ± 4.4	16.9 ± 5.9	< 0.0001
OHIP-14 score (out of 56)	11.3 ± 9.4 (*n* = 35)	7.1 ± 7.6	0.037
Salivary EGF output (pg/10 min)	9237.6 ± 8447.0	13,296.9 ± 7907.1	0.033

Mean ± SD

*SS* Sjögren’s syndrome, *OHIP-14* Oral Health Impact Profile-14

##### Comparison of salivary EGF levels in patients with and without SS

The salivary EGF output in the SS group (9237.6 ± 8447.0 pg/10 min) was significantly lower than that in the non-SS group (13,296.9 ± 7907.1 pg/10 min) (*p* = 0.033) (Table 1). Because the clinical background varied widely among the SS patients, the SS group was divided into two groups according to two clinical factors.

##### Disease duration

**Fig. 1:**
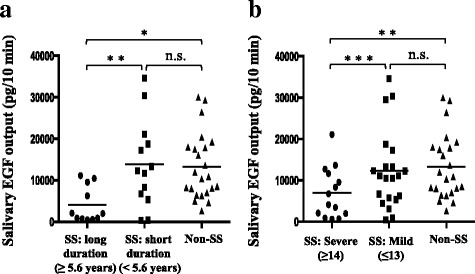
Salivary epidermal growth factor (EGF) levels of the Sjögren’s syndrome (SS) and non-SS groups. **a** The SS group was divided into the long-duration group and short-duration groups depending on disease duration, and salivary EGF output levels were compared between these groups and the non-SS group. **b** The SS group was divided into the severe and mild groups according to the severity of intraoral manifestations determined using the Oral Health Impact Profile (OHIP)-14 score, and salivary EGF output levels were compared between these groups and the non-SS group. Statistical differences were assessed using the Mann−Whitney *U* test. *p < 0.001, **p < 0.01, ***p < 0.05; n.s., not significant

The SS group was divided into the long- and the short-duration groups by disease duration. The cutoff level was provisionally set at 5.6 years based on the mean disease duration of the entire SS group (≥ 5.6 years: long-duration group [*n* = 11], < 5.6 years: short-duration group [*n* = 13]). The OHIP-14 score in the long-duration SS group (13.9 ± 10.8) was significantly higher than that in the non-SS group (7.1 ± 7.6) (*p* < 0.05), but the score did not differ significantly between the short-duration SS group and the non-SS group. With regard to the salivary flow rate, the rate was significantly lower in the long-duration SS group (4.7 ± 2.4 mL/10 min) than in the short-duration SS group (9.1 ± 5.7 mL/10 min) and the non-SS group (16.9 ± 5.9 mL/10 min) (*p* < 0.05 and *p* < 0.0001, respectively). The rate in the short-duration SS group was also significantly lower than that in the non-SS group (*p* < 0.001) (Table 2). Furthermore, the salivary EGF output in the long-duration SS group (4087.2 ± 4356.7 pg/10 min) was significantly lower than that in the short-duration SS group (13,881.3 ± 10,480.2 pg/10 min) and the non-SS group (13,296.9 ± 7907.1 pg/10 min) (*p* < 0.01 and *p* < 0.001, respectively). However, no significant difference was found in the salivary EGF output in the short-duration SS group compared to that in the non-SS group (Fig. 1a).

**Table 2:** Clinical characteristics of the SS and non-SS groups. Classification of the SS group by disease duration

	SS: long duration (≥ 5.6 years) (*n* = 11)	SS: short duration (< 5.6 years) (*n* = 13)	Non-SS (*n* = 23)
Disease duration (years)	9.2 ± 1.8*	2.6 ± 1.3	–
Age (years)	63.9 ± 5.9**	53.2 ± 13.0	56.1 ± 17.4
OHIP-14 score (out of 56)	13.9 ± 10.8^†^	8.6 ± 6.6 (*n* = 11)	7.1 ± 7.6
Salivary flow rate (mL/10 min)	4.7 ± 2.4***, ^†††^	9.1 ± 5.7^††^	16.9 ± 5.9

*p < 0.0001 versus the short duration (< 5.6 years) group, **p < 0.01 versus the short duration (< 5.6 years) group, ***p < 0.05 versus the short duration (< 5.6 years) group, ^†^*p* < 0.05 versus the non-SS group, ^††^*p* < 0.001 versus the non-SS group, ^†††^*p* < 0.0001 versus the non-SS group

##### Oral health-related quality of life (OHIP-14 score)

The SS group was stratified according to the OHIP-14 score into the severe intraoral manifestations group and the mild group. When one point out of four was given for all 14 questions, the total OHIP-14 score was 14. Therefore, the cutoff level was provisionally set at 14 points (≥ 14: severe group [*n* = 14], ≤ 13: mild group [*n* = 21]). In the severe SS group, the disease duration was longer and the salivary flow rate was lower than that in the mild SS group, but neither showed a significant difference (Table 3). The salivary EGF output in the severe SS group (6965.8 ± 6161.1 pg/10 min) was significantly lower than that in the mild SS group (12,275.7 ± 9420.0 pg/10 min) and the non-SS group (13,296.9 ± 7907.1 pg/10 min) (*p* < 0.05 and *p* < 0.01, respectively). In contrast, the salivary EGF output did not differ significantly between the mild SS group and the non-SS group (Fig. 1b).

**Table 3:** Clinical characteristics of the SS and non-SS groups. Classification of the SS group by oral health-related quality of life (OHIP-14 score)

	SS: severe (≥ 14) (*n* = 14)	SS: mild (≤ 13) (*n* = 21)	Non-SS (*n* = 23)
Age (years)	61.5 ± 10.4*	52.7 ± 14.0	56.1 ± 17.4
Disease duration (years)	7.2 ± 3.7 (*n* = 9)	4.9 ± 3.6 (*n* = 13)	–
Salivary flow rate (mL/10 min)	6.9 ± 4.2^†^	9.3 ± 4.4^†^	16.9 ± 5.9

Mean ± SD

*SS* Sjögren’s syndrome, *OHIP-14* Oral Health Impact Profile-14

*p < 0.05 versus the mild (≤ 13) group, ^†^*p* < 0.0001 versus the non-SS group

##### Correlation analysis

**Fig. 2:**
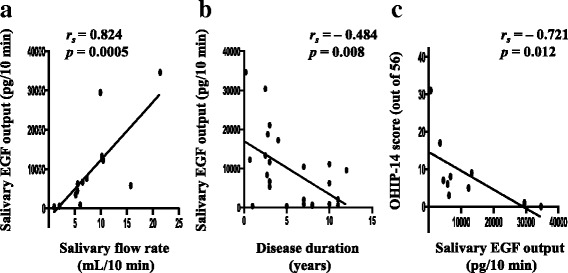
Correlations between different factors and salivary epidermal growth factor (EGF) output in the Sjögren’s syndrome (SS) group. **a** Correlation of salivary flow rate with salivary EGF output (*n* = 13). **b** Correlation of disease duration with salivary EGF output (*n* = 24). **c** Correlation of the Oral Health Impact Profile (OHIP)-14 score with salivary EGF output (*n* = 10). Correlations were assessed using Spearman’s rank correlation coefficient

The correlation between salivary flow rate and salivary EGF output was evaluated in 13 SS patients, excluding those under medical treatment that might have affected salivary flow rate (e.g., muscarinic M3 receptor agonist, corticosteroids, and immunosuppressants). The salivary flow rate was found to be significantly correlated with salivary EGF output (*r*_*s*_ = 0.824, *p* = 0.0005) (Fig. 2a). In SS patients whose disease duration could be confirmed (*n* = 24), the disease duration was found to be significantly and inversely correlated with salivary EGF output (*r*_*s*_ = − 0.484, *p* = 0.008) (Fig. 2b). A similar analysis was also conducted in 10 SS patients excluding those under the abovementioned medical treatments to test the correlation between the OHIP-14 score and salivary EGF output. The OHIP-14 score was significantly and inversely correlated with salivary EGF output (*r*_*s*_ = − 0.721, *p* = 0.012) (Fig. 2c).

#### Assessment of changes in the salivary EGF levels within 3-year follow-up in the same patients with SS

##### Comparison of clinical data at baseline and at re-evaluation in patients with and without SS

No significant differences in age and sex were observed between the SS group (mean age, 59.5 ± 12.7 years, 21 women and two men) and the non-SS group (59.9 ± 15.2 years, 10 women and four men) (*p* = 0.468 and *p* = 0.173, respectively). The mean SS disease duration at baseline (initial evaluation) was 5.5 years.

In the SS group, there were no significant differences in the number of patients treated with a muscarinic M3 receptor agonist (pilocarpine or cevimeline) and corticosteroids or immunosuppressants at baseline (12/23 [52%] and 6/23 [26%], respectively) and at re-evaluation (14/23 [61%] and 8/23 [35%], respectively) (*p* = 0.766 and *p* = 0.749, respectively). The OHIP-14 score at re-evaluation (12.6 ± 9.2) was significantly higher than that at baseline (10.2 ± 8.8) (*p* = 0.040), indicating a significant exacerbation of the OHRQoL. No significant differences were observed in the salivary flow rate at baseline (8.1 ± 5.3 mL/10 min) and at re-evaluation (7.4 ± 5.1 mL/10 min) (*p* = 0.149). However, the salivary EGF output at re-evaluation (8352.8 ± 7813.3 pg/10 min) was significantly lower than that at baseline (10,158.4 ± 9820.9 pg/10 min) (*p* = 0.032) (Table 4 (a)).

**Table 4:** Clinical characteristics at baseline (initial evaluation) and at re-evaluation 3 years later

	Baseline (initial evaluation)	3 years later (re-evaluation)	*p* value
(a) SS group (*n* = 23)			
OHIP-14 score (out of 56)	10.2 ± 8.8 (*n* = 22)	12.6 ± 9.2 (*n* = 22)	0.040
Salivary flow rate (mL/10 min)	8.1 ± 5.3	7.4 ± 5.1	0.149
Salivary EGF output (pg/10 min)	10,158.4 ± 9820.9	8352.8 ± 7813.3	0.032
(b) Non-SS group (*n* = 14)			
OHIP-14 score (out of 56)	9.1 ± 6.8	10.7 ± 9.4	0.169
Salivary flow rate (mL/10 min)	16.8 ± 6.7	16.7 ± 6.0	0.628
Salivary EGF output (pg/10 min)	13,623.1 ± 9546.2	11,904.9 ± 6995.4	0.184

Mean ± SD

*SS* Sjögren’s syndrome, *OHIP-14* Oral Health Impact Profile-14

In the non-SS group, no significant differences were observed in the OHIP-14 score, salivary flow rate, and salivary EGF output at baseline (9.1 ± 6.8, 16.8 ± 6.7 mL/10 min, and 13,623.1 ± 9546.2 pg/10 min, respectively) and at re-evaluation (10.7 ± 9.4, 16.7 ± 6.0 mL/10 min, and 11,904.9 ± 6995.4 pg/10 min, respectively) (*p* = 0.169, *p* = 0.628, and *p* = 0.184, respectively) (Table 4 (b)).

##### Changes in salivary flow rate and EGF output according to disease duration and oral health-related quality of life

The clinical background varied widely among the SS patients in the present study; thus, the SS group was subdivided into two groups depending on two clinical factors.

##### Disease duration

**Fig. 3:**
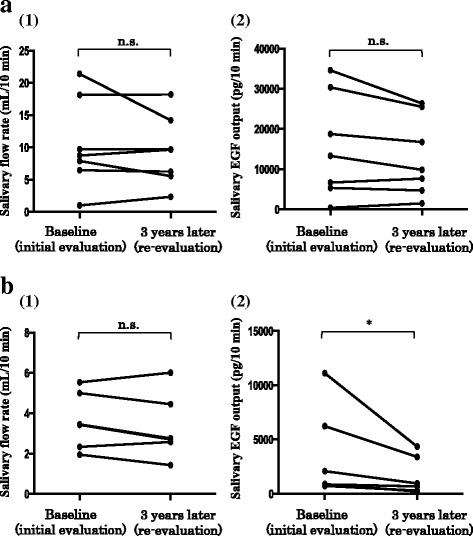
Changes in the salivary flow rate and salivary epidermal growth factor (EGF) output according to the disease duration. **a** Short-duration Sjögren’s syndrome (SS) group. **b** Long-duration SS group. (1) Changes in the salivary flow rate. (2) Changes in the salivary EGF output. Statistical analysis was performed using the Wilcoxon signed-rank test. *p < 0.05; n.s., not significant

The SS group was divided into short- and long-disease duration subgroups. The cutoff level was provisionally set at 5 years at the starting point based on the mean disease duration of the entire SS group (≤ 5 years: short-duration group [*n* = 7], ≥ 6 years: long-duration group [*n* = 6]). In the short-duration group, no significant differences were observed in the salivary flow rate and salivary EGF output between baseline (10.5 ± 7.0 mL/10 min and 15,646.9 ± 12,986.2 pg/10 min, respectively) and re-evaluation levels (9.4 ± 5.4 mL/10 min and 13,187.6 ± 9902.1 pg/10 min, respectively) (Fig. 3a). In contrast, in the long-duration group, although the salivary flow rate did not differ significantly between baseline (3.6 ± 1.4 mL/10 min) and re-evaluation levels (3.3 ± 1.6 mL/10 min), the salivary EGF output at re-evaluation (1640.9 ± 1774.8 pg/10 min) was significantly lower than that at baseline (3652.4 ± 4211.2 pg/10 min) (*p* < 0.05) (Fig. 3b).

##### Oral health-related quality of life (OHIP-14 score)

**Fig. 4:**
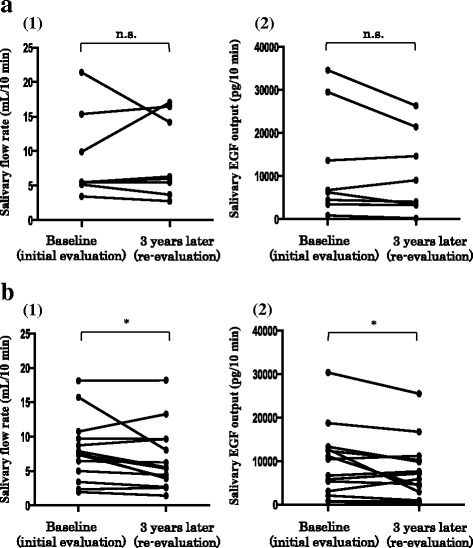
Changes in the salivary flow rate and salivary epidermal growth factor (EGF) output according to the extent of changes in the Oral Health Impact Profile (OHIP)-14 score at 3-year re-evaluation. **a** Patient group in which the OHIP-14 score decreased or did not change (non-exacerbation group). **b** Patient group in which the OHIP-14 score increased (exacerbation group). (1) Changes in the salivary flow rate. (2) Changes in the salivary EGF output. Statistical analysis was performed using the Wilcoxon signed-rank test. *p < 0.05; n.s., not significant

The SS group was subdivided on the basis of the magnitude of changes in the OHIP-14 scores after 3 years into the non-exacerbation group (the score decreased or was unchanged in 3 years; *n* = 8) and the exacerbation group (the score increased in 3 years; *n* = 14). In the non-exacerbation group, the salivary flow rate and the salivary EGF output did not significantly differ between baseline (9.0 ± 6.3 mL/10 min and 12,448.0 ± 12,727.1 pg/10 min, respectively) and re-evaluation values (9.0 ± 5.9 mL/10 min and 10,310.9 ± 9565.9 pg/10 min, respectively) (Fig. 4a). In contrast, in the exacerbation group, both the salivary flow rate and the salivary EGF output at re-evaluation (6.8 ± 4.6 mL/10 min and 7724.2 ± 6901.4 pg/10 min, respectively) were significantly lower than at baseline (8.0 ± 4.6 mL/10 min and 9548.6 ± 8063.6 pg/10 min, respectively) (*p* < 0.05 in both cases) (Fig. 4b).

## Decrease in salivary EGF levels: deterioration in saliva quality and intraoral manifestations in SS

The following novel results were obtained in the present study: (1) salivary EGF output in SS patients was significantly lower than that in non-SS patients. (2) In SS patients, with prolonged disease duration, in addition to the progressive reduction in the salivary flow rate, the salivary EGF output also decreased. The salivary EGF output correlated with the salivary flow rate and showed an inverse correlation with disease duration. (3) In cases of prolonged SS disease duration, the salivary EGF output in SS patients decreased more rapidly than the salivary flow rate in a short period of time. (4) The decrease in salivary EGF output as well as the salivary flow rate was closely associated with poor OHRQoL in SS patients. Koski et al. [22] reported that EGF expression diminished in the labial salivary glands of patients with SS and concluded that the continuous lymphocytic inflammation in SS affected not only the salivary flow rates but also the EGF production in the salivary glands. However, our reports are the first to demonstrate the association between SS and salivary EGF levels.

Hutson et al. [23] showed that wound healing of the skin was enhanced by licking, that is, by transfer of saliva to the wound. Subsequent reports have suggested that EGF synthesized in the salivary glands and secreted into the saliva is involved in wound healing inside and outside the oral cavity. In animal models, oral wound healing was delayed significantly after removal of the submandibular glands, which are the major source of salivary EGF in rodents, and oral administration or topical application of EGF was found to restore the rate of wound healing [10, 11]. Fujisawa et al. [11] reported that topical EGF application promoted proliferation of fibroblasts and keratinocytes and accelerated the healing of gingival ulcers. These findings suggest that salivary EGF is involved in repair mechanisms that lead to wound healing and maintenance of the integrity of the mucosa of the oral cavity.

Several studies have demonstrated an association between intraoral inflammatory diseases and changes in salivary EGF levels. Salivary EGF concentrations were found to be significantly lower in patients with aphthous stomatitis [4, 13] or peritonsillar abscess [4] and decreased even after healing and in the absence of these lesions [4, 13]. In patients with radiation-induced oral mucositis, salivary EGF levels were significantly lower and inversely correlated with the severity of oral mucositis [14, 15]. The authors of the above reports all speculated that low salivary EGF levels reduced the capacity of the oral mucosa to heal and maintain physiologic integrity, thereby increasing susceptibility to intraoral inflammatory lesions [4, 13–15].

Patients with SS frequently develop refractory intraoral inflammatory lesions, such as oral mucositis and glossitis. In the present study, the comparisons between various subgroups of SS patients showed a close association between lower salivary EGF levels and poorer OHRQoL in patients with SS. This finding indicated that decreased salivary EGF levels, that is, deterioration in saliva quality accompanied by lower intraoral clearance owing to hyposalivation, could play a role in the pathogenesis of refractory intraoral manifestations and reduced OHRQoL. Moreover, in the present study, the OHRQoL and salivary EGF output but not the salivary flow rate decreased significantly in the same patients with SS only 3 years after the initial evaluation. Such findings were not observed in patients without SS. This decrease was especially striking in SS patients with long disease duration and progressive exacerbation of the OHRQoL. These findings strongly support the association between intraoral manifestations and decreased salivary EGF levels in SS and suggest that salivary EGF output decreases rapidly within a short period of time and progressive worsening of the OHRQoL depends on this rapid decrease in salivary EGF output when the SS disease duration becomes prolonged.

## Topical EGF supplementation: a novel target for the therapeutic intervention of SS?

The currently accepted treatment for xerostomia in SS consists of maintaining good oral hygiene (e.g., mouthwash), using saliva substitutes (e.g., artificial saliva), and utilizing salivary stimulation (e.g., muscarinic M3 receptor agonists). The main purpose of these agents is to compensate for the reduced or inadequate supply of water via the natural saliva. Unfortunately, a substantial number of SS patients with refractory intraoral manifestations suffer from poor OHRQoL despite these treatments, especially in cases with long disease duration and declining residual salivary gland function. The findings of the present study and previous reports suggest that improving the quality of saliva by EGF supplementation into the oral cavity in combination with conventional treatments may promote mucosal healing, reduce the severity of intraoral manifestations, and ameliorate the OHRQoL in patients with SS. In previous studies using oral epithelial cell lines, the cell migration response [24] and the wound-closure effects [25] of EGF have been demonstrated. Girdler et al. [26] investigated the effect of an EGF mouthwash on oral ulceration in patients undergoing cancer chemotherapy. Although the rate of healing of established ulcers in patients who received EGF mouthwash and placebo did not differ, a delay in the onset and a smaller mean area of ulceration were noticed in the EGF mouthwash group. Patients develop oral mucosal manifestations rapidly in a few days after the initiation of chemotherapy. In SS, the progression of oral mucosal manifestations is not rapid compared with that as a consequence of chemotherapy. Considering the difference between the pathological mechanisms of both, we expect that topical EGF application will be more effective for SS patients than for patients undergoing chemotherapy. In addition, the EGF concentration was found to be decreased in the tear fluid of patients with SS [27, 28]. Tsubota et al. [29] reported that corneal epithelial damage decreased significantly after the initiation of treatment with autologous serum eye drops containing EGF, vitamin A, and transforming growth factor-β. These results strongly indicate that topical EGF application in the treatment of oral mucosal manifestations in patients with SS has not only an improving effect by EGF supplementation in patients with prolonged disease duration but also a prophylactic effect in patients with short disease duration. We expect that topical EGF application that can adhere to wounds longer than mouthwash (e.g., in the form of gel formulation) may lead to better results. However, the concentration and frequency of EGF administration remain to be elucidated. Excess EGF stimulates the proliferation and differentiation of malignant cells [30].

## Conclusions

A decrease in salivary flow rates and salivary EGF output appears with the progression of SS. The deterioration in saliva quality as well as lower intraoral clearance by hyposalivation could contribute to the progression of the intraoral manifestations. We believe that our findings may lead to the development of novel effective therapies for SS.

## Abbreviations

EGF: Epidermal growth factor
OHIP: Oral Health Impact Profile
OHRQoL: Oral health-related quality of life
QoL: Quality of life
SS: Sjögren’s syndrome

## References

[1] Stewart CM, Berg KM, Cha S, Reeves WH (2008). Salivary dysfunction and quality of life in Sjögren syndrome: a critical oral-systemic connection. J Am Dent Assoc.

[2] Zelles T, Purushotham KR, Macauley SP, Oxford GE, Humphreys-Beher MG (1995). Saliva and growth factors: the fountain of youth resides in us all. J Dent Res.

[3] Cohen S (1962). Isolation of a mouse submaxillary gland protein accelerating incisor eruption and eyelid opening in the new-born animal. J Biol Chem.

[4] Ino M, Ushiro K, Ino C, Yamashita T, Kumazawa T (1993). Kinetics of epidermal growth factor in saliva. Acta Otolaryngol Suppl.

[5] Heitz PU, Kasper M, van Noorden S, Polak JM, Gregory H, Pearse AG (1978). Immunohistochemical localisation of urogastrone to human duodenal and submandibular glands. Gut.

[6] Thesleff I, Viinikka L, Saxén L, Lehtonen E, Perheentupa J (1988). The parotid gland is the main source of human salivary epidermal growth factor. Life Sci.

[7] Ino M, Ushiro K, Ino C, Yamashita T, Kumazawa T, Takahashi T. Epidermal growth factor in salivary glands—epidermal growth factor and its receptor in human salivary glands—. Jibirinsho. 1992;85:805–14. (in Japanese).

[8] Scaltriti M, Baselga J (2006). The epidermal growth factor receptor pathway: a model for targeted therapy. Clin Cancer Res.

[9] Nakamura H, Kawakami A, Ida H, Koji T, Eguchi K (2007). EGF activates PI3K-Akt and NF-κB via distinct pathways in salivary epithelial cells in Sjögren’s syndrome. Rheumatol Int.

[10] Noguchi S, Ohba Y, Oka T (1991). Effect of salivary epidermal growth factor on wound healing of tongue in mice. Am J Phys.

[11] Fujisawa K, Miyamoto Y, Nagayama M (2003). Basic fibroblast growth factor and epidermal growth factor reverse impaired ulcer healing of the rabbit oral mucosa. J Oral Pathol Med.

[12] Playford RJ (1995). Peptides and gastrointestinal mucosal integrity. Gut.

[13] Adişen E, Aral A, Aybay C, Gürer MA (2008). Salivary epidermal growth factor levels in Behçet’s disease and recurrent aphthous stomatitis. Dermatology.

[14] Dumbrigue HB, Sandow PL, Nguyen KH, Humphreys-Beher MG (2000). Salivary epidermal growth factor levels decrease in patients receiving radiation therapy to the head and neck. Oral Surg Oral Med Oral Pathol Oral Radiol Endod.

[15] Epstein JB, Gorsky M, Guglietta A, Le N, Sonis ST (2000). The correlation between epidermal growth factor levels in saliva and the severity of oral mucositis during oropharyngeal radiation therapy. Cancer.

[16] Azuma N, Katada Y, Kitano S, Sekiguchi M, Kitano M, Nishioka A (2014). Correlation between salivary epidermal growth factor levels and refractory intraoral manifestations in patients with Sjögren’s syndrome. Mod Rheumatol.

[17] Azuma N, Katada Y, Kitano S, Sekiguchi M, Kitano M, Nishioka A (2015). Rapid decrease in salivary epidermal growth factor levels in patients with Sjögren’s syndrome: a 3-year follow-up study. Mod Rheumatol.

[18] Fujibayashi T, Sugai S, Miyasaka N, Hayashi Y, Tsubota K (2004). Revised Japanese criteria for Sjögren’s syndrome (1999): availability and validity. Mod Rheumatol.

[19] Vitali C, Bombardieri S, Jonsson R, Moutsopoulos HM, Alexander EL, Carsons SE (2002). European study group on classification criteria for Sjögren’s syndrome. Classification criteria for Sjögren’s syndrome: a revised version of the European criteria proposed by the American-European Consensus Group. Ann Rheum Dis.

[20] Slade GD (1997). Derivation and validation of a short-form oral health impact profile. Community Dent Oral Epidemiol.

[21] Yamazaki M, Inukai M, Baba K, John MT (2007). Japanese version of the Oral Health Impact Profile (OHIP-J). J Oral Rehabil.

[22] Koski H, Konttinen YT, Hietanen J, Tervo T, Malmöstrom M (1997). Epidermal growth factor, transforming growth factor-alpha, and epidermal growth factor receptor in labial salivary glands in Sjögren’s syndrome. J Rheumatol.

[23] Hutson JM, Niall M, Evans D, Fowler R (1979). Effect of salivary glands on wound contraction in mice. Nature.

[24] Royce LS, Baum BJ (1991). Physiologic levels of salivary epidermal growth factor stimulate migration of an oral epithelial cell line. Biochim Biophys Acta.

[25] Oudhoff MJ, Bolscher JG, Nazmi K, Kalay H, van’t Hof W, Amerongen AV (2008). Histatins are the major wound-closure stimulating factors in human saliva as identified in a cell culture assay. FASEB J.

[26] Girdler NM, McGurk M, Aqual S, Prince M (1995). The effect of epidermal growth factor mouthwash on cytotoxic-induced oral ulceration. A phase I clinical trial. Am J Clin Oncol.

[27] Pflugfelder SC, Jones D, Ji Z, Afonso A, Monroy D (1999). Altered cytokine balance in the tear fluid and conjunctiva of patients with Sjögren’s syndrome keratoconjunctivitis sicca. Curr Eye Res.

[28] Ohashi Y, Ishida R, Kojima T, Goto E, Matsumoto Y, Watanabe K (2003). Abnormal protein profiles in tears with dry eye syndrome. Am J Ophthalmol.

[29] Tsubota K, Goto E, Fujita H, Ono M, Inoue H, Saito I (1999). Treatment of dry eye by autologous serum application in Sjögren’s syndrome. Br J Ophthalmol.

[30] Tokunaga A, Onda M, Okuda T, Teramoto T, Fujita I, Mizutani T (1995). Clinical significance of epidermal growth factor (EGF), EGF receptor, and c-erbB-s in human gastric cancer. Cancer.

